# Supplementary material for: Deletion of wfs1 Impairs Oligodendrocyte Precursor Cells Dorsal Distribution and Myelination Through the wfs1-hmgcs1 Axis in Zebrafish
Source: Biology (Basel). 2026 Aug 16;15(16):1402. doi: 10.3390/biology15161402 (PMC13510151; doi:10.3390/biology15161402)
Supplement: Supplementary file 1 [file biology-15-01402-s001.zip › File S1-Transcriptome sequencing data and differentially expressed genes (DEGs) in wfs1-- zebrafish at 5 dpf/2.KEGG/wfs1KOvsWT/DOWN/src/dre00512.html]

dre00512


- dre:568500

- Down regulated genes

568500(-3.74523561261366)

- dre:557675

- Down regulated genes

557675(-6.94923938358647)

- dre:324510

- Down regulated genes

324510(-1.44577671953651)

- dre:568500

- Down regulated genes

568500(-3.74523561261366)

- dre:568500

- Down regulated genes

568500(-3.74523561261366)

- dre:568500

- Down regulated genes

568500(-3.74523561261366)

- dre:568500

- Down regulated genes

568500(-3.74523561261366)

- dre:568500

- Down regulated genes

568500(-3.74523561261366)

- dre:557675

- Down regulated genes

557675(-6.94923938358647)

- dre:324510

- Down regulated genes

324510(-1.44577671953651)

- dre:568500

- Down regulated genes

568500(-3.74523561261366)

- dre:557675

- Down regulated genes

557675(-6.94923938358647)

- dre:568500

- Down regulated genes

568500(-3.74523561261366)

- dre:568500

- Down regulated genes

568500(-3.74523561261366)

- dre:568500

- Down regulated genes

568500(-3.74523561261366)

- dre:557675

- Down regulated genes

557675(-6.94923938358647)

- dre:568500

- Down regulated genes

568500(-3.74523561261366)

- dre:568500

- Down regulated genes

568500(-3.74523561261366)

- dre:557675

- Down regulated genes

557675(-6.94923938358647)

Close
